# Separating Patients with SEID from Those with CFS in the French ME/CFS Association, with Some Thoughts on Nomenclature

**DOI:** 10.3390/diagnostics12051095

**Published:** 2022-04-27

**Authors:** Julien Campagne, Isabelle Fornasieri, Barbara Andreani, Monique Eginard, Jean-Dominique de Korwin

**Affiliations:** 1 Internal Medicine Department, University of Lorraine, 34, Cours Leopold, CS 25233, CEDEX, 54052 Nancy, France; julien.campagne@uneos.fr; 2 Internal Medicine Department, University Hospital of Nancy, Rue du Morvan, CEDEX, 54511 Vandœuvre-Lès-Nancy, France; 3 Faculty of Psychology, University of Strasbourg, 12, Rue Goethe, 67000 Strasbourg, France; isabelle.fornasieri@unistra.fr; 4 French Association for Chronic Fatigue Syndrome (ASFC), Maison des Associations Nice Centre, 3 bis, rue Guigonis, 06300 Nice, France; 5 Regional Center for Scientific Documentation and Clinical Research, Legouest Army Instruction Hospital, 27, Avenue de Plantières, 57077 Metz, France; credorc@hia-legouest.fr; 6 French Association for Chronic Fatigue Syndrome (ASFC), 25, Impasse des Lavandes, 13710 Fuveau, France; me.asso.sfcf@gmail.com

**Keywords:** systemic exertion intolerance disease, chronic fatigue syndrome, myalgic encephalomyelitis, case definition, patient association, patient opinion

## Abstract

In 2015, the American Institute of Medicine, now called the National Academy of Medicine, (IOM/NAM) proposed new diagnostic criteria for both Myalgic Encephalomyelitis/Chronic Fatigue Syndrome (ME/CFS) and a new label: Systemic Exertion Intolerance Disease (SEID). This study aimed to evaluate the SEID criteria among members of the French Association of ME/CFS (ASFC) and their opinion about this new name. We sent an anonymous questionnaire to 494 ASFC members, using French-translated questions derived from the IOM/NAM tool kit. Among the 178/231 responding subjects who reported ME/CFS diagnosis, 150 (84%) met the criteria of SEID. For each set of questions, we identified some of them that significantly distinguished SEID from non-SEID patients concerning unrefreshing sleep, cognitive disorders, and orthostatic intolerance items. Forty-six percent of the respondents considered the “SEID” terminology as more appropriate than “CFS”, 39% considered it inappropriate, and 15% had no opinion. Some questions better identified the SEID criteria. The IOM/NAM SEID criteria captured a large part of ASFC members suffering from ME/CFS. However, this new SEID label was not well accepted by the subjects, nor were the other denominations, suggesting that a better term should be found. Pending development of specific markers, further work with patient communities is needed to find a more suitable label.

## 1. Introduction

The term ‘Chronic Fatigue Syndrome’ (CFS) was firstly used by Holmes et al. in 1988 to rename chronic Epstein–Barr virus syndrome [1]. In 1994, Fukuda et al. redefined the CFS criteria—these are still commonly used [2]. Myalgic Encephalomyelitis (ME) was initially described separately from CFS by clinicians and researchers [3,4]. Then, Carruthers et al. placed both conditions under the umbrella term “ME/CFS” and proposed the Canadian Clinical Criteria (CCC) of ME/CFS [5]. In 2011, an International Consensus Panel considered that ME was the more appropriate name for ME/CFS and redefined the previous criteria introducing symptom severity [6]. Nevertheless, these criteria are complex and difficult to use in routine clinical practice, leaving patients and physicians distraught [7]. Debate continues on the differences between ME and CFS [8]. Thus, the Institute of Medicine (IOM), now called the National Academy of Medicine, (NAM) convened a Committee on the Diagnosis Criteria for ME/CFS in 2015 to develop evidence-based diagnostic criteria to help clinicians [9]. The Committee finally recommended a new label for ME/CFS in order to more accurately capture the central characteristics of the illness: Systemic Exertion Intolerance Disease (SEID). Fewer criteria are needed than for ME: for a positive diagnosis at least 4/5 core symptoms should present at least half the time with moderate or severe intensity, including 3 mandatory criteria (fatigue > 6 months with substantial impairment in all activities, unrefreshing sleep, post-exertional malaise), and cognitive impairment and/or orthostatic intolerance [9]. Other diagnoses, such as fibromyalgia (FM), were considered as comorbidities. Overlapping syndromes are usually considered in different classifications [6]. While the name “CFS” is controversial among patients because it can convey a negative impression of laziness [10], “ME” is preferred because of its imputation of real, inflammatory disease, which is not completely demonstrated but supported by some studies [11,12,13].

ME/CFS is a complex, chronic medical condition affecting multiple body systems and its pathophysiology is still being investigated. In European countries, especially in France, ME/CFS remains insufficiently known by general practitioners [7,14]. For patients, obtaining medical care can be a real obstacle with a high likelihood of delayed diagnosis [7,9,14]. They can suffer from epistemic injustice since they are harmed specifically in their capacity as knowers [15]. Thus, there is a strong demand for more simple and suitable criteria for routine practice. The ME/CFS French patients’ association (ASFC) was approved by the Health Ministry in 2015 (www.asso-sfc.org, accessed on 27 January 2022). The association welcomes everyone suffering from unexplained chronic fatigue and informs and refers them to specialized centers to get an accurate diagnosis. Then, ASFC proposes a follow-up of members/patients with regular meetings with volunteers from everywhere in France. ASFC also organizes an annual meeting with expert scientists to present research and the latest advances in the field.

The aims of this study were to assess the suitability of the new SEID criteria in ASFC members by using a questionnaire derived from the practical recommendations proposed by the IOM/NAM [9], define the more disabling symptoms, and seek their views on the new “SEID” terminology.

## 2. Materials and Methods

### 2.1. Survey of ASFC Members

The ASFC association comprises 494 members who are most often diagnosed CFS or ME after clinical, biological, and imaging exams to eliminate other causes of fatigue (chronic infections, endocrine disorders, autoimmune diseases, depression, etc.). We sent each member a specific questionnaire by private mail to determine if they met the SEID criteria and get their opinion on the new name SEID. The questionnaire was completely anonymous with return by mail or to the local committees of the ASFC association (paper version). The patients gave their consent by sending the answers to the questionnaire, but without giving their identity. The questionnaires were then numbered by the ASFC association and forwarded to the investigators. Data were collected over 9 months. All questionnaires were examined.

### 2.2. Questionnaire

The questionnaire was built by the ASFC Scientific Committee, composed of ME/CFS French experts, and previously tested by five randomly chosen members of ASFC. They assessed that questions, which were translated by a professional, were clear, understandable, and truly corresponding to their feelings. Demographic factors (age, sex) and fatigue characteristics (duration, onset, triggering factors) were collected. Subjects had to declare one or more current diagnosis (CFS, ME, FM, or self-added proposals). The SEID criteria were evaluated by questions derived from the practical recommendations proposed by the IOM/NAM [9], giving examples of terms ME/CFS patients commonly use to describe their symptoms and potential questions that can alert clinicians to the diagnosis: disabling fatigue (3 questions), Post-Exertional Malaise (PEM) (4 questions), unrefreshing sleep (3 questions), cognitive impairment (9 questions), and orthostatic intolerance (4 questions) (Supplementary Table S1). In order to evaluate the frequency and severity, subjects had to report if their symptoms were present more or less than 50% of the time, or if they were missing entirely, as proposed by IOM/NAM [9]. We recorded some other symptoms, which were used previously in Fukuda’s case definitions [2] and cited as additional symptoms for SEID [9]: generalized pain, sore throat, swollen cervical or axillary lymph nodes, cold sensation, and unusual sweats. Then, they had to rate six symptoms (pain, activity limitation, PEM, unrefreshing sleep, cognitive impairment, and orthostatic intolerance) from 1 to 6 according to their disabling impact. Finally, subjects were asked to state if the new denomination of SEID seemed more suitable to characterize their conditions and to comment on its impact in familial, medical, social, and professional environments, using a Likert four modalities scale. Free comments were also possible and were analyzed.

### 2.3. SEID Diagnosis

We excluded individuals aged <18 years and those who did not declare chronic fatigue. We also excluded subjects with alternative medical or psychiatric conditions or another condition such as fibromyalgia that could explain their ME/CFS symptoms. An SEID criterion was adopted in case of a positive answer to one or more symptoms-related questions and if the symptom was present at least 50% of the time. According to the diagnostic criteria for ME/CFS proposed by the IOM/NAM [9], to be classified as an SEID patient, respondents had to meet the three mandatory criteria (disabling fatigue, PEM, unrefreshing sleep) and at least one of the two facultative criteria (cognitive impairment or orthostatic intolerance). The distinctive value of symptom questions used to identify the different criteria for SEID diagnosis was analyzed.

### 2.4. Symptoms Ranking

We asked subjects to rate six symptoms (pain, activities limitation, PEM, unrefreshing sleep, cognitive impairment, and orthostatic intolerance) from 1 to 6 according to their disabling impact (Rank 1 for the most disabling, Rank 6 for the least). To display these observations, we built a radar chart that consists of a sequence of equi-angular spokes, with each spoke representing a rank. The data length of a spoke was proportional to the magnitude of the variable (proportion of subjects who classified the symptom at this rank). For each symptom, a line was drawn connecting the data values for each spoke (i.e., each rank). The star-like appearance of the plot enabled comparison of the disabling impact of each symptom.

### 2.5. Statistical and Textual Analysis

We used Epi Info™ software to analyze the data. To compare the two subgroups, SEID and non-SEID, we performed a non-parametric Mann–Whitney test for quantitative variables and a Chi-squared test for continuous variables. The scores, which were measured on the Likert scale, characterized the patient opinions and were calculated for the whole population. The text data were assessed by a semantic analysis of the key ideas associated with the SEID denomination.

## 3. Results

**Figure 1:**
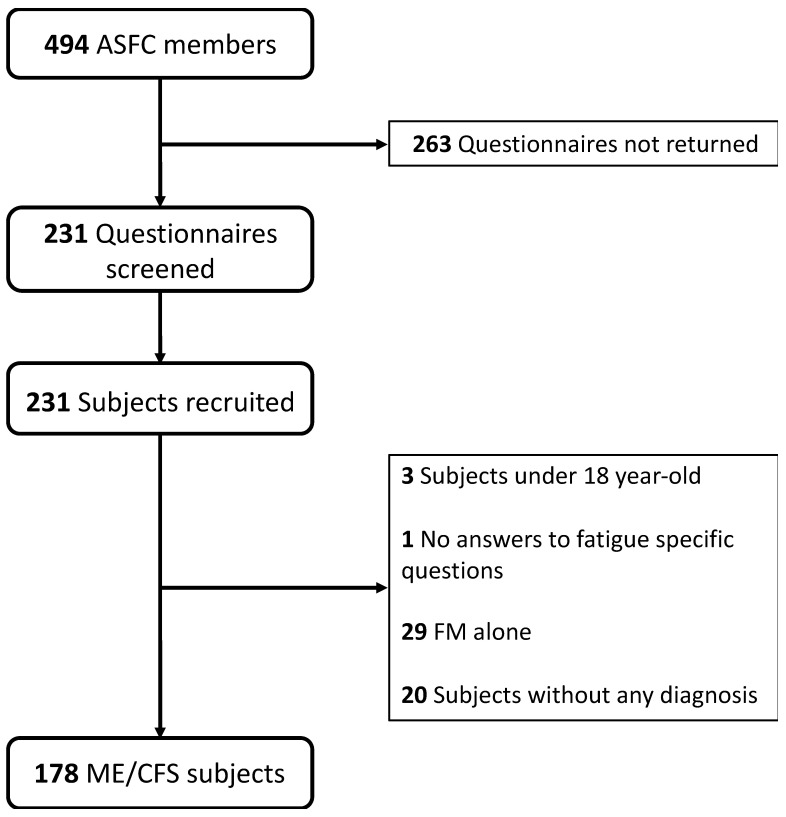
Study flowchart: from the 494 members of ASFC, 231 patients were recruited and after selection, 178 were included as they suffered from ME/CFS.

Two-hundred and thirty-one of the four-hundred and ninety-four members returned the questionnaire. Four subjects were excluded (3 were <18 years-old, 1 did not experience chronic fatigue). Twenty subjects with no medical diagnosis and 29 declaring fibromyalgia alone were excluded. CFS alone was diagnosed in 134 subjects, ME alone in 10 subjects, and both conditions in 34 subjects. Thus, SEID status was evaluated in 178 ME/CFS subjects (Figure 1).

### 3.1. Demographics 

All individuals declared that fatigue had lasted for more than 6 months with substantial impairment. The entire population was aged 51.7 years on average and mostly composed of women (84%) (Table 1). Fatigue onset was almost equally sudden (47%) or progressive (53%). Among the 104 individuals who reported one or more triggers of fatigue, a majority declared an infection (69%), followed by psychological causes (13%) such as breakdown or burn-out, then surgery (8%) and hormonal disorders (7%) such as hypothyroidism. The delay between fatigue onset and diagnosis was substantial: 7 years on average. Regarding the medical specialization of the clinician who made the diagnosis (data not shown), internists were in the front line (33%), followed by rheumatologists (26%) and general practitioners (20%). Twenty percent of the patients have consulted two or more specialists. Concerning the other symptoms associated with fatigue in Fukuda’s CFS classification, generalized pain, sore throat, swollen cervical lymph nodes, swollen axillary lymph nodes, cold sensation, and unusual sweats were declared by 83%, 48%, 36%, 11%, 73%, and 61% of the subjects, respectively.

### 3.2. SEID vs. Non-SEID Subjects

In the 178 ASFC members who reported ME/CFS diagnosis, 150 (84%) met the criteria of SEID. The characteristics of SEID and non-SEID patients (Table 1) did not differ concerning age, sex, fatigue onset, trigger, fatigue diagnosis, and other symptoms: generalized pain, sore throat, swollen lymph nodes, cold sensation, and unusual sweats. Only diagnostic delay was significantly different: 7.2 years for SEID patients versus 5.3 years for non-SEID patients.

### 3.3. Distinctive Value of Symptom Questions for SEID Diagnosis 

Concerning SEID criteria in the entire population, chronic fatigue, PEM, unrefreshing sleep, cognitive impairment, and orthostatic intolerance were experienced by 100%, 98%, 87%, 85%, and 93%, respectively. Unrefreshing sleep, cognitive impairment, and orthostatic intolerance were more frequently reported in the SEID than in the non-SEID group (*p* < 0.01) (Table 2). In “fatigue” criteria, no IOM/NAM symptom questions were distinctive, contrary to “PEM” with a strong difference between SEID and non-SEID patients for 3 out of 4 proposals: “I feel crashed, relapsed, collapsed”, “I feel mentally tired after the slightest effort”, and “I feel physically drained or sick after mild activity” (*p* < 0.001). For “unrefreshing sleep”, the 3 IOM/NAM symptom questions (“I feel like I never slept”, “I cannot fall asleep or stay asleep”, and “After long or normal hours of sleep, I still don’t feel good in the morning”) distinguished SEID patients from non-SEID (*p* < 0.001). For cognitive impairment, the difference was less clear with only 2 out of 9 questions separating SEID or non-SEID patients: “It is hard to concentrate, I cannot focus” (68% vs. 39%, *p* < 0.001), “I cannot do several tasks at the same time” (68% vs. 48%, *p* < 0.01). For the other optional criterion, “orthostatic intolerance”, no symptom question was distinctive.

### 3.4. Disability Degree of Each Symptom in SEID Group 

**Figure 2:**
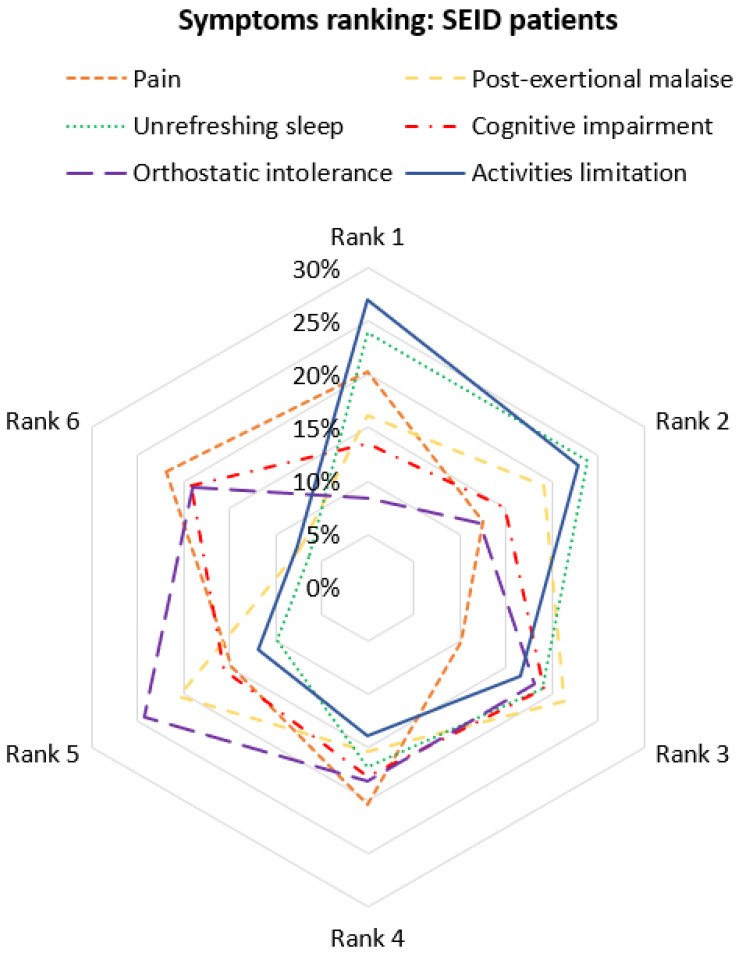
Radar chart of the most disabling symptoms rating for SEID patients.

For patients meeting SEID criteria, limitation of activities, unrefreshing sleep, and pain were rated as the most disabling factor by more than one-fifth of the patients (27%, 24%, and 20% at rank 1, respectively) (Figure 2). At rank 2, unrefreshing sleep (24%) and activities limitation (23%) remained the most disabling factors, followed by PEM (19%).

### 3.5. Respondents’ Opinion of SEID Label 

Forty-six percent (28% slightly) of respondents declared that “SEID” terminology is more appropriate than “CFS”, 39% answered not at all, and 15% had no opinion (Table 3 (A)). The majority of subjects thought it would give a negative image of the disease to their family (56%), to medical staff or social workers (54%), and to colleagues at work (57%). Almost all subjects answered these questions except for the one about the image of their disease “at work” (*r =* 122, 81%).

We analysed the free comments of subjects to understand the reasons for their point of view (Table 3 (B)). In negative comments, 23% of subjects considered that “exertion intolerance” was a highly negative expression referring to “laziness” or “effort allergy”. It was pointed out by 16% of them that their fatigue does not occur only after exertion but without any effort or for minimal tasks. This new name would not help in social life with their family, friends, colleagues, and practitioners (11%). SEID does not reflect other symptoms (9%) and is too complicated (9%), especially with the use of “systemic”, which could not be understood by the general public (9% versus 2% supportive). On the contrary, the use of “disease” instead of “syndrome” was appreciated in positive comments (16%). Some people spontaneously made proposals to use terms that would seem more appropriate, such as keeping “myalgic encephalomyelitis” alone, using “exhaustion” instead of “exertion intolerance”, and including handicap or disability level, chronicity, or even keeping “chronic fatigue syndrome”.

## 4. Discussion

### 4.1. Population Studied

This study is an evaluation of SEID criteria proposed by IOM/NAM [9] by questioning all members of the French CFS Association (ASFC) who experienced unexplained fatigue. Almost 50% of them (231/494) returned the questionnaire, suggesting high motivation to participate in the survey. A major part of them declared they suffered from ME/CFS and we assumed that they met a specialist who performed relevant explorations to give a diagnosis. As with the population studied in the current study, ME/CFS patients in general are known to be mostly women [16,17,18]. Interestingly, when subjects could identify a fatigue trigger, most of them reported an infection. The role of microbiological agents in pathophysiology of ME/CFS has been studied, particularly with viral infections [19]. Hickie et al. observed a relatively uniform post-infective fatigue syndrome persisting in a significant minority of patients for six months or more after clinical infection with several different viral and non-viral micro-organisms, including EBV [20]. Katz el al. reported the development of CFS in the follow-up of adolescent girls with infectious mononucleosis [21]. Diagnostic delay was 7 years on average, underlining the difficulties encountered by patients and physicians in this field. The French situation is worse than in the US where 67% to 77% of patients have reported 1 year to get a diagnosis, and 29% reported longer than 5 years [9].

### 4.2. Main Symptoms

Pain remains an important feature of ME/CFS. In our survey, subjects who declared FM alone were excluded, but 83% of subjects with ME/CFS suffered from pain. Meeus et al. reviewed the medical literature and concluded that chronic musculoskeletal pain was a widespread occurrence in ME/CFS patients [22]. A major part of those fulfilling Fukuda et al. criteria demonstrated muscle or joint pain (94% and 84%, respectively) [22,23]. Cold perception in ME/CFS patients has not been studied much. Of note, cold limbs were reported in two previous studies by 50% and 66% of ME/CFS patients, respectively [24,25]. Unusual sweats is another neuroendocrine manifestation, which is experienced by more ME/CFS patients than healthy controls [25]. Like “immunological” symptoms (sore throat, swollen lymph nodes), the IOM/NAM considered that evidence was insufficient to include these symptoms in SEID criteria [9].

### 4.3. SEID Diagnosis

Eighty-four percent of the selected subjects met the SEID criteria. Jason et al. have already tested these new criteria in self-reported ME, CFS, or ME/CFS patients recruited in tertiary cares of US, Great Britain, and Norway, and captured 88% of 796 participants [26], which is comparable to the 92% that met the Fukuda criteria and close to our result, but also identifying a larger group of patients than the Canadian ME/CFS and ME criteria. In a survey on early symptoms during ME/CFS, Chu et al. also concluded that SEID criteria, requiring fewer symptoms, categorized a similar percentage of subjects (72%) as the Fukuda criteria (79%) or the CCC (71%), whereas the ME-ICC categorized a significantly lower percentage of subjects (61%, *p* < 0.01) [27]. Nevertheless, Jason et al. disagreed with their main conclusion that the percentage of patients selected by the IOM/NAM criteria is comparable to the percentage captured by other research case definitions [28]. This debate illustrates differences of opinion about how case definitions are operationalized, but SEID criteria seems to be well-matched with the recent findings, such as mild neuro-inflammation and lower levels of metabolites [29].

### 4.4. SEID vs. Non-SEID

In our study, SEID and non-SEID patients had similar characteristics, except diagnosis delay was longer in SEID subjects (7.2 vs. 5.3 years). This result has no clear explanation. However, in Fukuda’s criteria, which is the most used in France [7], PEM, cognitive disorders, and unrefreshing sleep are optional criteria [2]. In our study, these symptoms were less frequently reported in non-SEID patients. The SEID criteria have been proposed recently and seem to be frequently used by new experts in France due to their ease of use for clinical diagnosis. This could explain the later diagnosis in patients with previously unexplained chronic fatigue.

By comparing the weight of the questions suggested by the IOM/NAM between the two groups a posteriori, we could emphasize some of them.

#### 4.4.1. PEM

PEM is an exacerbation of ME/CFS symptoms that occurs after physical or cognitive exertion and leads to a reduction in functional ability [5]. The prevalence of PEM among ME/CFS patients as diagnosed by the existing criteria varies from 69% to almost 100% [24,25,30]. This symptom did not distinguish our SEID and non-SEID subjects, affecting 86–100% of them. PEM remains difficult to assess: the way PEM is defined can affect how patients interpret the concept of PEM and whether they endorse it [8,31,32], and PEM was recently shown to be composed of two empirically different experiences, one for generalized fatigue and one for muscle-specific fatigue [33]. Three out of four of the IOM/NAM suggested symptoms questions (“I feel crashed, relapsed, collapsed”, “I feel mentally tired after the slightest effort”, and “I feel physically drained or sick after mild activity”) [9] distinguished SEID and non-SEID patients. The last one (“The more demanding or prolonged the activity, the more severe and prolonged the payback”) was the only one that was not distinctive. An explanation could be that PEM occurs with minimal effort, thus it is impossible to grade for more intense exercises. This “crash experience” has often had a delayed onset after the effort and is prolonged for hours and days.

#### 4.4.2. Unrefreshing Sleep

Unrefreshing sleep or feeling tired upon waking or before going to bed, is among the most common symptoms reported by ME/CFS patients, and only a small percentage of patients diagnosed with ME/CFS failed to report some type of sleep dysfunction. The three IOM/NAM symptom questions were discerning between SEID and non-SEID patients, highlighting the central role of this symptom.

The IOM/NAM Committee decided that only cognitive impairment and/or orthostatic intolerance criteria is required for SEID diagnosis, without any clear justification [9].

#### 4.4.3. Cognitive Impairment

Numerous studies demonstrated high rates of neurological symptoms in ME/CFS: 69% to 93% for attention deficit, 80% to 85.6% for memory disturbance, and 73% to 75.5% for difficulties with words [25,30], for instance. Only 2 of 9 symptom questions we used separated SEID from non-SEID patients: “It is hard to concentrate, I cannot focus” and “I cannot do several tasks at the same time”. Of the 9 proposals, these two symptoms have the strongest impact in daily life, with a substantial reduction in functioning, which is a mandatory condition for SEID diagnosis [9].

#### 4.4.4. Orthostatic Intolerance

Orthostatic intolerance is defined as a clinical condition in which symptoms worsen upon assuming and maintaining upright posture and are ameliorated (although not necessarily abolished) by recumbency [34,35]. It is a common feature of ME/CFS patients [24,36,37,38,39,40]. Interestingly, cognitive impairment and orthostatic intolerance were experienced by a high proportion of subjects in the current study.

No questions about orthostatic intolerance were discerning, whereas the overall symptom was. The explanation was for the number of available data in each group: 8 non-SEID patients did not respond to this part and the remaining 20 had at least one symptom and met the criteria. Thus, we considered that only 71% (20/28) experienced orthostatic intolerance. We similarly analyzed SEID patients with 145 respondents of 150 total (97%).

If only cognitive impairment was required, 17 individuals (11%) would not have fulfilled IOM/NAM criteria, whereas if orthostatic intolerance had been the only mandatory criterion, 5 subjects (3%) would not have been captured. In a ME/CFS population, Jason et al. found a lower proportion of orthostatic intolerance (67%) than cognitive impairment rate (93%) or other SEID criteria [26]. In this work, experiencing orthostatic intolerance or cognitive impairment enabled 2% more participants to meet SEID criteria than if only cognitive impairment was needed [26]. A recent study evaluated Postural Orthostatic Tachycardia Syndrome (POTS), which is included in orthostatic intolerance syndromes, by an active standing test and blood pressure measurement in a population of CFS patients [41]. In these patients, but also in those meeting the SEID criteria (76%), the proportion of POTS remained very low (5.7% and 5.2%), thus POTS did not seem to be a good ME/CFS marker [41]. Concerning IOM/NAM proposals to assess orthostatic intolerance, no question discriminated SEID from non-SEID patients in our study.

#### 4.4.5. Symptoms’ Ranking

Subjects had to rank different symptoms according to their disability level. Apart from the “limitation of activities”, which is the consequence of all the others, pain appeared as one of the most impairing symptoms. For patients who meet 2003 or 2010 ICC criteria, pain was already described as more severe and damaging [8,23,42]. This result underlines the place of pain as a core symptom in SEID patients with a strong effect on daily life.

#### 4.4.6. Mixed Opinion about “SEID” Label

Only a small proportion of subjects reported having no opinion, except for the impact on relations at work (27%). One explanation could be that the disease often leads to unemployment [43,44].

Huibers & Wessely considered that one of the many controversies surrounding chronic fatigue syndrome is the possible impact of the diagnostic label: is it disabling or enabling? [45]. The answer to the question of ‘to label or not to label’ may turn out to depend not on the label, but on what that label implies. “SEID” terminology was considered slightly or entirely more appropriate than “CFS” by 46% of respondents, compared to 39% who said it was not at all appropriate. Only 12 (9%) people argued to keep “ME”, whereas 29 proposed another term and 29 (23%) found the term exertion intolerance pejorative. Neither the term SEID nor those of CFS and ME were considered ideal, indicating that a search for a better term was desirable.

Free comments were often harsh, reflecting the distress of these individuals [46]. For patients, “exertion intolerance” could be misunderstood as “fatigue” with the connotation that it is a patient’s fault still. More than half of the ME/CFS subjects worried about the image SEID could present of their disease to their family, with medical staff or social workers, and with colleagues. Petrison, from the Paradigm Change organization, interviewed 1004 people, including 89% of ME/CFS patients, about the idea of using the term SEID as a replacement for the label ME/CFS [47]. Around 62% of participants said the proposed terminology was pretty bad or very bad. Jason et al. reported on an international sample of 1045 participants and 65% of the US sample and 68% of the international sample liked or definitely liked the term ME, whereas only 16% to 17% liked or definitely liked SEID [48]. The name “Chronic Fatigue Syndrome” has been largely criticized by patients. It leads relatives or colleagues, and even clinicians, to think the illness is not real [10] and that patients are lazy and just need to exercise more, which is dangerous and overly simplistic yet common advice. Johnson et al. studied the risk factors for suicide in ME/CFS patients and showed that those who utilized the CFS label were more likely to die of suicide [49].

Conversely, the common opinion in the patient community is that “myalgic encephalomyelitis” suggests a more neurological and physiological illness—not only psychological [47,50,51]. In a survey from the Solve ME/CFS Initiative, a patient-research organization, members were asked about their preferences for illness labels [52]. Most of them (55%) preferred the term ME. Twisk proposed renouncing SEID to keep ME for a neurological-based condition, and replace CFS, the fatigue-based condition, by one or more meaningful, non-stigmatizing names [53,54].

### 4.5. Limitations

There were several limitations to the current study. Our method could have introduced a selection bias as very severe patients may not have been able to complete the questionnaire. It is known that these patients need more support during the research process [55]. This survey only reported the opinion of the members of an association of ME/CFS patients who are probably the most motivated. We also did not collect entire medical histories. Medical diagnoses were evaluated only by self-reporting, without confirmation of the real clinician diagnosis, but we supposed that self-reported diagnoses were accurate as ASFC executives refer all members to ME/CFS specialists to obtain confirmation of the diagnosis.

A limitation comes from the current debate on SEID investigation. While the IOM/NAM do recommend the development of a toolkit appropriate for screening and diagnosing patients with ME/CFS, it does not actually detail the contents of this toolkit. As part of this toolkit, the IOM/NAM even feel that the development of clinical questionnaires or history tools that are valid across populations of patients should be an urgent priority [9]. Rigorous recommendations when applying SEID diagnostic criteria were investigated by Asprusten et al. with an adolescent CFS cohort [56]. This study used variables from a total of eight validated questionnaires to operationalize the SEID criteria, and then used baseline data to decide whether a patient fulfilled those criteria or not. Two CFS subgroups (SEID vs. non-SEID) were compared across baseline characteristics, as well as a wide range of cardiovascular, inflammatory, infectious, neuroendocrine, and cognitive variables. In the absence of a specific questionnaire for the identification of SEID criteria, we created an original questionnaire based on the recommendations of the IOM/NAM for identification of symptoms in patients who are characteristic of SEID. The questions were formulated using the usual expressions of patients identified by the IOM/NAM [9], and we did not find during the preliminary test and survey any difficulties in comprehension or interpretation. Further symptom surveys comparing SEID criteria and existing ME/CFS case definitions operationalized the case definition criteria using the DePaul Symptom Questionnaire symptom scale (DSQ) to obtain most symptoms covered by the different classifications [26,27]. However, DSQ has not been validated in the French framework yet.

From perspectives on etiology and pathophysiology, ME/CFS has been labeled differently, which influenced changes in case definitions and terminologies [29]. All definitions have their limits in the current absence of validated diagnostic criteria other than clinical ones. Due to the insufficiency of objective markers—and, in particular, biological markers—ME/CFS effectively remains a syndrome with all the uncertainties concerning its precise definition and the possibility of subgroups of different causes or mechanisms.

We excluded 29 subjects reporting FM alone, which was associated with fatigue but without ME/CFS as a declared diagnosis. Similar to the IOM/NAM [9], we grouped CFS and ME under the umbrella label ME/CFS. ME diagnosis was rarely reported alone (10 subjects, data not shown), which is not enough to be analyzed as an independent subgroup. This choice was criticized by Twisk [53,54], who considered that CFS and ME were partially overlapping but clearly distinct conditions according to their case definitions [2,57]. The CFS main criteria is unexplained chronic fatigue, whereas ME needs typical neuro-muscular signs [53]. The PEM criterion is also mandatory as in both the ME and SEID classifications [6,9]. The recent update of the NICE recommendations indicates that four symptoms must be present for the diagnosis of ME/CFS, which are included in the diagnostic criteria of SEID: fatigue, post-exertional malaise, unrefreshing sleep, and cognitive difficulties [58]. Other symptoms including orthostatic intolerance and neuromuscular symptoms may also be associated but are not exclusive to ME/CFS [58].

Early studies into long COVID symptomatology suggest many overlaps with clinical presentation of ME/CFS. Advancements in and standardization of long COVID research methodologies would improve the quality of future research and may allow further investigations into the similarities and differences between long COVID and ME/CFS [59].

## 5. Conclusions

This study is the first in France to assess the symptoms of patients declaring that they have ME/CFS. It showed that patients from the ME/CFS French patients’ association (ASFC) met the criteria for Systemic Exercise Intolerance Disease (SEID) using questions derived from the practical recommendations proposed by the IOM/NAM. Since some SEID criteria could be more precisely investigated by appropriate questions focusing on exploring different components of the condition, we believe that a self-reported questionnaire such as ours was useful as a screening instrument to check the patient’s complaints. We identified several distinguishing questions to assess each criteria. These findings could lead to a more concise set of questions to make SEID diagnosis. Even if a change of name for ME/CFS is expected, less than half the concerned patients thought the SEID label was appropriate and the majority of them worried about the negative image this name would present in different social environments. Neither the term ME nor that of SEID was considered ideal, only the conclusion to search for a better term seems supported. Without objective markers, the illness label remains controversial. Patients’ comments showed a need for future debates to get an acceptable and representative name.

## Figures and Tables

**Table 1:** Characteristics of all subjects and comparison between SEID and non-SEID patients. *r* number of respondents to each item; (%); *ns*, not significant.

	ALL	SEID	NON SEID	*p* Value
**Population**	**n = 178**	**n = 150**	**n = 28**	
**Age**	*r* = 178	*r* = 150	*r* = 28	
Mean	51.7	51.7	51.7	*ns*
**Sex**	*r* = 178	*r* = 150	*r* = 28	
Women	149 (84)	127 (85)	22 (79)	*ns*
Men	29 (16)	23 (15)	6 (21)	
**Fatigue onset**	*r* = 137	*r* = 118	*r* = 19	
Sudden	64 (47)	52 (44)	12 (63)	*ns*
Progressive	73 (53)	66 (56)	7 (37)	
**Trigger**	*r* = 104	*r* = 82	*r* = 22	
Infectious	72 (69)	56 (68)	16 (73)	*ns*
Psychological	14 (13)	9 (11)	5 (23)	*ns*
Surgery	8 (8)	7 (9)	1 (5)	*ns*
Hormonal disorder	7 (7)	7 (9)	0 (0)	*ns*
**Diagnostic delay**	*r* = 159	*r* = 135	*r* = 24	
Mean	7.0	7.2	5.3	5.04 × 10^−4^
**Other symptoms**	*r* = 174	*r* = 149	*r* = 25	
Generalized pain	144 (83)	124 (83)	20 (80)	*ns*
Sore throat	83 (48)	71 (48)	12 (48)	*ns*
Swollen cervical lymph nodes	62 (36)	50 (34)	12 (48)	*ns*
Swollen axillary lymph nodes	19 (11)	16 (11)	3 (12)	*ns*
Cold sensation	127 (73)	106 (71)	21 (84)	*ns*
Unusual sweats	106 (61)	92 (62)	14 (56)	*ns*

**Table 2:** Comparison of each question suggested by the IOM/NAM to assess SEID criteria between SEID and non-SEID patients. *r*, number of respondents to each item; (%); *ns*, not significant.

SEID Criteria	SEID		Non SEID	
**1-Fatigue**	***r =* 150**	**150 (100)**	***r =* 28**	**28 (100)**
*S1. Compared to what you were able to do before being exhausted,*				
P1. I feel a flu-like fatigue/exhaustion	*r =* 149	80 (54)	*r =* 28	17 (61)
P2. I feel like a battery that is never able to be recharged fully despite resting a lot and limiting my activities	*r =* 150	142 (97)	*r =* 28	25 (89)
P3. Thinking takes a lot more work than it used to	*r =* 150	108 (72)	*r =* 28	20 (71)
**2-Post-exertional malaise**	***r =* 150**	**150 (100)**	***r =* 28**	**24 (86)**
*S2. After a physical or mental activities, prolonged standing,*				
P1. I feel crashed, relapsed, collapsed	*r =* 150	142 (95)	*r =* 28	20 (71)
P2. I feel mentally tired after the slightest effort	*r =* 146	95 (65)	*r =* 28	7 (25)
P3. I feel physically drained or sick after mild activity	*r =* 149	136 (91)	*r =* 28	16 (57)
P4. The more demanding or prolonged the activity, the more severe and prolonged the payback	*r =* 150	144 (96)	*r =* 28	24 (86)
**3-Unrefreshing sleep**	***r =* 150**	**150 (100)**	***r =* 28**	**4 (14)**
*S3. Concerning my sleep,*				
P1. I feel exhausted like I never slept	*r =* 148	128 (86)	*r =* 28	3 (11)
P2. I cannot fall asleep or stay asleep	*r =* 146	123 (84)	*r =* 28	3 (11)
P3. After long or normal hours of sleep, I still don’t feel good in the morning	*r =* 148	119 (80)	*r =* 27	3 (11)
**4-Cognitive impairment**	***r =* 150**	**133 (89)**	***r =* 28**	**19 (68)**
*S4. On the intellectual level or to carry out certain activities such as driving, reading a book, watching a movie, working on computer or participating in a discussion,*				
P1. I feel like a brain fog	*r =* 146	73 (50)	*r =* 28	10 (36)
P2. I feel confused	*r =* 143	57 (40)	*r =* 28	7 (25)
P3. I feel disoriented	*r =* 144	47 (33)	*r =* 28	4 (14)
P4. It is hard to concentrate, I cannot focus	*r =* 148	101 (68)	*r =* 28	11 (39)
P5. I cannot process information	*r =* 146	46 (32)	*r =* 28	5 (18)

**Table 3:** Opinion of SEID patients about SEID terminology and the image given by this new name in different social areas (A). Free comments analysis (B). *r*, number of respondents for each item; (%).

(A)	
SEID	
*n* = 150	
**Is SEID terminology more appropriate than CFS?**	*r* = 150
Absolutely	27 (18)
Slightly	42 (28)
Not at all	59 (39)
I don’t know	22 (15)
**Problem with the image its gives of the disease?**	
**In your family?**	*r* = 149
*Very concerned*	45 (30)
*Slightly concerned*	39 (26)
*Not concerned*	44 (30)
*I don’t know*	21 (14)
**In social relationships?**	*r* = 147
*Very concerned*	44 (30)
*Slightly concerned*	35 (24)
*Not concerned*	36 (24)
*I don’t know*	32 (22)
**At work?**	*r* = 122
Very concerned	46 (38)
Slightly concerned	23 (19)
Not concerned	20 (16)
I don’t know	33 (27)
**(B)**	
**Unfavorable comments**	
*"Exertion intolerance" is pejorative*	29 (23)
*means laziness, patients are "shiftless"*	
*Fatigue without exertion/with minimal effort*	21 (16)
*no better professional/administrative/ medical recognition*	14 (11)
*More than just an "exertion intolerance"*	12 (9)
*Too complicated, "systemic" not understood*	11 (9)
*Not "serious" or "scientific" enough*	7 (5)
*Removal of "chronic" term*	3 (2)
**Favorable comments**	
*Use of "disease" term*	21 (16)
*Use of "systemic" term*	3 (2)
*Removal of "fatigue" term*	2 (2)
**Name proposals**	
*Keep "ME"*	12 (9)
*Use of "exhaustion" term*	10 (8)
*Reflect handicap/disability*	6 (5)
*Include chronic pain*	4 (3)
*Keep "CFS"*	3 (2)
*Use "FM"*	3 (2)
*Include cognitive signs,*	3 (2)
*refer to biological causes, use a proper name, use "syndrome", "neurological", "multisystemic", "immunological", "disabling" terms, a more scientific name*	

## Data Availability

Internal Medicine Department, University of Lorraine, 34, Cours Leopold, CS 25233, CEDEX, 54052 Nancy, France; julien.campagne@uneos.fr.

## Supplementary Materials

The following supporting information can be downloaded at: https://www.mdpi.com/article/10.3390/diagnostics12051095/s1, Table S1: Evaluation of SEID criteria, extracted from the questionnaire, based on IOM/NAM proposals.
