# Supplementary material for: Separating Patients with SEID from Those with CFS in the French ME/CFS Association, with Some Thoughts on Nomenclature
Source: Diagnostics (Basel). 2022 Apr 27;12(5):1095. doi: 10.3390/diagnostics12051095 (PMC9139646; doi:10.3390/diagnostics12051095)
Supplement: Supplementary file 1 [file diagnostics-12-01095-s001.zip › diagnostics-1673719-supplementary.pdf]

Supplementary Table S1. Evaluation of SEID criteria, extracted from the questionnaire, based on IOM/NAM proposals.

Mutiple choice questions.
Please, select the proposal closest to what you feel.

1- *Fatigue*
Compared to what you were able to do before being exhausted,
- I feel a flu-like fatigue/exhaustion
  Never Less than 50% of time More than 50% of time
- I feel like a battery that is never able to be recharged fully despite resting a lot and limiting my activities
  Never Less than 50% of time More than 50% of time
- Thinking takes a lot more work than it used to
  Never Less than 50% of time More than 50% of time

2- *Post-exertional malaise*
After a physical or mental activities, prolonged standing,
- I feel crashed, relapsed, collapsed
  Never Less than 50% of time More than 50% of time
- I feel mentally tired after the slightest effort
  Never Less than 50% of time More than 50% of time
- I feel physically drained or sick after mild activity
  Never Less than 50% of time More than 50% of time
- The more demanding or prolonged the activity, the more severe and prolonged the payback
  Never Less than 50% of time More than 50% of time

3- *Unrefreshing sleep*
Concerning my sleep,
- I feel exhausted like I never slept
  Never Less than 50% of time More than 50% of time
- I cannot fall asleep or stay asleep
  Never Less than 50% of time More than 50% of time
- After long or normal hours of sleep, I still don't feel good in the morning
  Never Less than 50% of time More than 50% of time

4- *Cognitive impairment*
On the intellectual level or to carry out certain activities such as driving, reading a book, watching a movie, working on computer or participating in a discussion,
- I feel like a brain fog
  Never Less than 50% of time More than 50% of time
- I feel confused
  Never Less than 50% of time More than 50% of time
- I feel disoriented
  Never Less than 50% of time More than 50% of time
- It is hard to concentrate, I cannot focus
  Never Less than 50% of time More than 50% of time
- I cannot process information
  Never Less than 50% of time More than 50% of time
- I cannot find the right words
  Never Less than 50% of time More than 50% of time
- I cannot do several tasks at the same time

Never Less than 50% of time More than 50% of time
- I cannot do a choice, make a decision
Never Less than 50% of time More than 50% of time
- I am absent-minded, forgetful
Never Less than 50% of time More than 50% of time

5- *Orthostatic intolerance*
When I get up or when I have been standing for a long time,
- I feel lightheadedness
Never Less than 50% of time More than 50% of time
- I feel unwell
Never Less than 50% of time More than 50% of time
- I feel dizziness, fainting
Never Less than 50% of time More than 50% of time
- Symptoms get better if I lie down and/or lift my legs
Never Less than 50% of time More than 50% of time
